# Hyperammonemia Associated with Valproic Acid Concentrations

**DOI:** 10.1155/2014/217269

**Published:** 2014-04-29

**Authors:** Marta Vázquez, Pietro Fagiolino, Cecilia Maldonado, Ismael Olmos, Manuel Ibarra, Silvana Alvariza, Natalia Guevara, Laura Magallanes, Ivette Olano

**Affiliations:** ^1^Pharmaceutical Sciences Department, Faculty of Chemistry, Universidad de la República, Avenida General Flores 2124, P.O. Box 1157, 11800 Montevideo, Uruguay; ^2^Therapeutic Drug Monitoring Service, “Dr. Manuel Quintela” Clinical Hospital, Universidad de la República, Avenida Italia s/n, 11609 Montevideo, Uruguay; ^3^“Sanatorio Canzani” Hospital, Banco de Previsión Social, Martín García 1363, 11800 Montevideo, Uruguay

## Abstract

Valproic acid, a branched short-chain fatty acid, has numerous action mechanisms which turn it into a broad spectrum anticonvulsant drug and make its use possible in some other pathologies such as bipolar disorder. It is extensively metabolized in liver, representing **β**-oxidation in the mitochondria one of its main metabolic route (40%). Carnitine is responsible for its entry into the mitochondria as any other fatty acid. Long-term high-dose VPA therapy or acute VPA overdose induces carnitine depletion, resulting in high levels of ammonia in blood. As a high correlation between salivary valproic acid levels and plasma ultrafiltrate levels was found in humans, saliva becomes a promising monitoring fluid in order to study valproic acid pharmacokinetics and its toxic effect. Extended-release (twice daily) formulations of valproic acid or carnitine supplementation are the proposed two therapeutic strategies in order to reverse hyperammonemia.

## 1. Introduction

The main goal of clinical pharmacokinetics is to enhance drug efficacy and decrease drug toxicity of a patient's therapy. The understanding of pharmacokinetics faces many problems. Genetics, age, gender, and disease determinants among others can affect drug absorption and disposition [1–3]. The interplay of these factors determines drug concentrations over time and the effect at the action site. Too little drug exposure leads to ineffective regimens, whereas too much creates the risk of adverse effects. Pathology itself can be the cause of pharmacokinetic variability [4–6]. Finally, although drug concentrations at the different sites are always responsible for the therapeutic or toxic effects observed, we have to be aware of the concentrations we are referring to: parent drug, metabolite, and free plasma drug concentration.

Valproic acid (VPA), a branched short-chain fatty acid, is believed to affect the function of the neurotransmitter GABA in the human brain. Its principle mechanism of action is believed to be the inhibition of the transamination of GABA (by inhibiting GABA transaminase) and there is substantial evidence that valproate increases GABA synthesis and release [7]. However, several other mechanisms of action have been proposed in recent years. VPA also blocks the voltage-gated sodium channels and T-type calcium channels [8]. These mechanisms make VPA a broad spectrum anticonvulsant drug and it is also prescribed for the treatment of bipolar disorder, schizoaffective disorder, social phobias, neuropathic pain, and for the prophylaxis and treatment of migraine headaches [9, 10].

It is highly protein bound to albumin (87–95%) and extensively metabolized in liver. Focusing on this last issue, there are at least 3 routes of VPA metabolism in humans: glucuronidation, *β*-oxidation in the mitochondria (both considered major routes accounting for 50% and 40% of dose, resp.), and *ω*-oxidation (considered a minor route, approximately 10%) but leading, the latter, to the formation of a toxic metabolite (4-en-VPA) [8, 11]. VPA crosses the membrane of liver mitochondria via the facilitation of carnitine. This pathway consists of several steps until valproylcarnitine is formed and in the mitochondrial matrix turns into valproyl-CoA which is able to get into the *β*-oxidation process. However, long-term high-dose VPA therapy or acute VPA overdose induces carnitine depletion and this could increase the *ω*-oxidation route with higher concentration of 4-en-VPA [12]. All this could result in incorrect ammonium elimination through urea cycle. Toxic metabolites of VPA inhibit carbamoyl phosphate synthetase (CPS), which catalyzes the conversion of ammonia to carbamoyl phosphate in the first step of the urea cycle [13]. Moreover, impairment of the beta-oxidation pathway produces acetyl-CoA depletion leading to a decreased synthesis of N-acetyl glutamic acid, an allosteric activator of CPS. Altogether these actions provide the basis for the development of hyperammonemia that could lead to encephalopathy. Hyperammonemia symptoms (seizures or neurological dysfunctions) are difficult to differentiate from the pathology itself (epilepsy or bipolar disorder) and they can be misdiagnosed as therapeutic failure instead of an adverse event related to the use of VPA. Central nervous system (CNS) symptoms can range from mild drowsiness and confusion to significant encephalopathy and coma resulting from cerebral edema. Ammonia excess is toxic to the CNS. As the brain does not produce urea from ammonia, its removal relies almost exclusively on glutamine synthetase, basically localized in astrocytes. The metabolism of ammonia to glutamine appears to be necessary and is followed by an osmotic disturbance in the brain, mitochondrial dysfunction with oxidative stress, and alterations in brain glucose metabolism. Cerebral blood flow is also altered and strongly influences the development of brain edema and intracranial hypertension [10, 14–21]. Besides, ammonia can increase levels of glutamate [22] resulting in activation of N-methyl-D-aspartate (NMDA) receptors. This is probably the cause of seizures.

Carnitine deficiency could also cause an impairment of other fatty acids oxidation leading to the obesity and lipids disorders that some VPA-treated patients experience [23].

Therapeutic drug monitoring is commonly carried out using plasma drug concentrations (free plus protein bound drug). But as it is known, this concentration does not usually represent the concentration at its receptor site. Only free drug can reach the biophase and interact with a receptor to produce the effect (therapeutic or toxic). Total drug concentrations depend on protein binding and this factor plays an important role in monitoring a drug with high affinity to albumin as is the case of VPA. Although monitoring of free drug levels is advisable, it could be time consuming or rather expensive for routine practice.

Our group has been working intensively with salivary concentrations of anticonvulsants [24–26]. For a number of drugs, mainly those which are lipophilic and nonionized at salivary pH range (i.e., phenytoin or carbamazepine), studies have demonstrated that salivary concentrations highly correlate with free drug concentration in plasma and therefore are more reflective of the concentration in the biophase [24, 25]. If drug diffusion into saliva is only passive, the factors involved in drug excretion to this fluid are its lipophilicity and the degree of ionization. The latter depends on drug pKa (4.8 for valproic acid) and its relationship with salivary and blood pHs. So VPA is more ionized in plasma than in saliva, and hence lower concentrations would be found in this fluid than those free in plasma.

Saliva VPA monitoring has been criticized [27, 28] as saliva/plasma ratio (S/P) shows a high variance. VPA variable protein binding and different salivary pHs could be responsible for the variability found as well as differences in saliva volumes [26]. Variability in saliva drug concentration could be diminished by using stimulated saliva (parafilm chewing or crystals of citric acid on the tongue). Otherwise, without stimulation, variable volume of saliva could determine higher variability. Bearing all this in mind, S/P ratios should be lower than free plasma/total plasma drug ratio.

The aim of the study was in the first place (a) to compare free plasma drug concentrations obtained by plasma ultrafiltration to salivary levels, and in the second place (b) to give a pharmacological insight of the possible concentration-dependent mechanism involved in hyperammonemia reaction. For this purpose, VPA and 4-en-VPA plasma and saliva levels correlations with ammonia levels in patients are going to be shown and thirdly (c) to analyze possible therapeutic measures that can be taken in order to reverse hyperammonemia.

## 2. Materials and Methods

### 2.1. Saliva-Plasma Ultrafiltrate Correlation

Eleven children diagnosed with epilepsy and receiving VPA monotherapy were included in the study. Saliva and blood samples (prior to the morning dose) were withdrawn. Blood was collected by arm venipuncture and drawn into heparinized tubes. For saliva collection, citric acid crystals were placed on the tongue and similar volumes from all the patients (1.5 mL) were collected.

Blood samples obtained from the patients were used to measure unbound and total plasma concentrations. Blood and saliva samples were centrifuged and were stored at −20°C until analysis. Analyses of total, free plasma, and saliva VPA concentrations were performed by fluorescence polarization immunoassay (FPIA, AxSYM, Abbott Laboratories). Free VPA drug was obtained by ultrafiltration using a SIGMA 3 K 18 refrigerated centrifuge and Centrifree Micropartition System (Amicon-Centrifree). One milliliter of plasma was pipetted into the device, centrifuged at 2000 g at 25°C ± 2°C for 20 minutes. Plasma albumins were determined by colorimetric analysis (Hitachi 911 Automatic Analyser, Roche Laboratories). Salivary pHs were obtained by Henderson-Hasselbalch equation:

(1) SP=[(1+10(pHsaliva−pKa))(1+10  (pHplasma−pKa))]×fpfs

with fp and fs being the unbound fractions in plasma and saliva, respectively.

### 2.2. Correlations between VPA or 4-en VPA Levels and Ammonia Levels in blood

Sixteen adult patients between 18 and 55 years old were included in the study. Patients were either epileptic or suffer bipolar disorders. Saliva and blood samples were withdrawn and stored under the same conditions as study 2.1.

Saliva and plasma VPA and plasma 4-en-VPA determinations were performed by a previously published validated high-performance liquid chromatography method (HPLC) with ultraviolet-visible (UV-Vis) [29] with minor modifications. Thirty microliters of internal standard (octanoic acid, OCT) was added to 1.0 mL of plasma or saliva. A Phenomenex Luna CN 5 *μ*m (150 mm × 4.6 mm) column was used as stationary phase. The mobile phase was a mixture of potassium phosphate monobasic 40 mM pH 3.4/acetonitrile (90/10) pumped with a flow rate of 1.5 mL/min. The column compartment was kept at 36°C, and the wavelength detection was 210 nm. Under these conditions the retention times of analytes were 3.8, 4.9, and 6.8 min for 4-en VPA, VPA, and OCT, respectively. The HPLC method was linear between 1.1 mg/L and 11 mg/L for VPA in saliva, between 1.1 mg/L and 133 mg/L for VPA in plasma, and between 0.78 mg/L and 16.5 mg/L for 4-en VPA in plasma. Within-day and between-day precisions (coefficient of variation: CV) for low, intermediate, and high concentrations of the different curves were below 15%. Accuracies at the same concentration levels were within 92 and 108%.

Ammonia concentration in blood was determined by Hitachi 911 Automatic Analyser, Roche Laboratories.

Pearson's correlation analysis was performed for the correlation of the variables.

### 2.3. Pharmacokinetic Parameters and Therapeutic Measures

Two female patients taking VPA were included. (i) One patient, with bipolar disorder, was receiving 1500 mg of VPA delayed-release (Valcote 500 mg, Abbott Laboratories) per day (1000 mg at 8:00 a.m. and 500 mg at 8:00 p.m.) for more than two weeks and then was switched to an extended-release dosage form marketed in our country (DIDPA-LP 500 mg, Athena Laboratories) with the same dosage schedule. On both occasions, salivary samples (every two hours) were collected during the 12-hour interval in order to study the drug level profiles, and ammonia concentrations were also performed. Pharmacokinetic parameters were obtained at steady state (ss): area under the saliva VPA concentration-time curve from 0 to *T* hours (AUC_ss  0–*T*_) calculated by the linear trapezoidal rule with *T* being the administration interval, experimental maximum and minimum concentration (*C*max_ss_ and *C*min_ss_), of the curve, time to obtain maximum concentration (*T*max_ss_), mean concentration (*C*mean_ss_ = AUC_ss  0–*T*_/*T*) and peak-trough fluctuation [PTF = (*C*max_ss_–*C*min_ss_) × 100/*C*mean_ss_].

(ii) The other patient, with epilepsy, was receiving 1500 mg of VPA per day (500 mg every 8 hours). Saliva and plasma VPA and plasma 4-en-VPA determinations as well as ammonia levels were performed. Due to an increased ammonia level, one gram daily of L-carnitine was administered orally for two months and the same determinations were carried out.

## 3. Results and Discussion

All plasma albumin concentrations for the different studies were within the normal range (3.3–5.0 g/dL).

**Figure 1:**
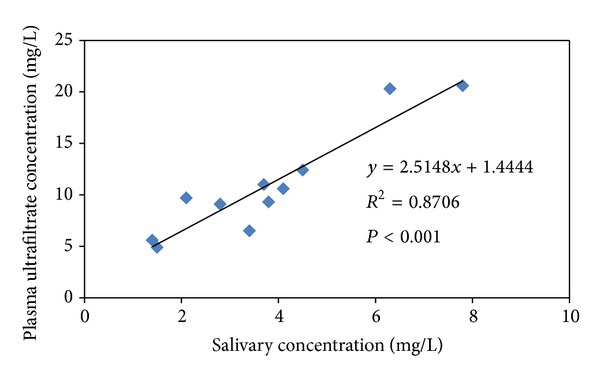
VPA ultrafiltrate concentration versus salivary VPA levels.

Table 1 shows VPA levels in plasma, plasma ultrafiltrate, and saliva as well as salivary pHs obtained from Henderson-Hasselbalch equation. As it can be observed from the table, stimulation with citric acid makes salivary pHs less variable among subjects (CV = 1.4%) since the salivary portion obtained is more closely correlated with the arterial free plasma drug concentration than with the vein one [26]. Moreover, S/P ratio is lower than the ultrafiltrate/plasma (ULT/P) ratio due to the degree of ionization of VPA (*p*Ka = 4.8). Salivary pHs were calculated according to Henderson-Hasselbalch equation and this could be not completely valid as this equation is only used with passive diffusion of drugs and active diffusion can also take place as efflux pumps are expressed at the apical membrane of both ductal and acinar cells of salivary glands [30]. Although, according to some authors [31], VPA appeared not to be a substrate of P-glycoprotein (P-gp) mediated efflux transporters or multidrug resistance associated proteins (MRPs), this issue is still under discussion. Anyway, citric stimulation seems to be adequate in order to diminish pH variability. Interestingly, as it is shown in Figure 1, salivary concentrations of VPA highly correlate with VPA ultrafiltrate concentrations. This makes saliva an extremely useful tool as monitoring fluid for studying therapeutic and toxic effects of VPA as it correlates with the free drug responsible for these effects.

**Figure 2:**
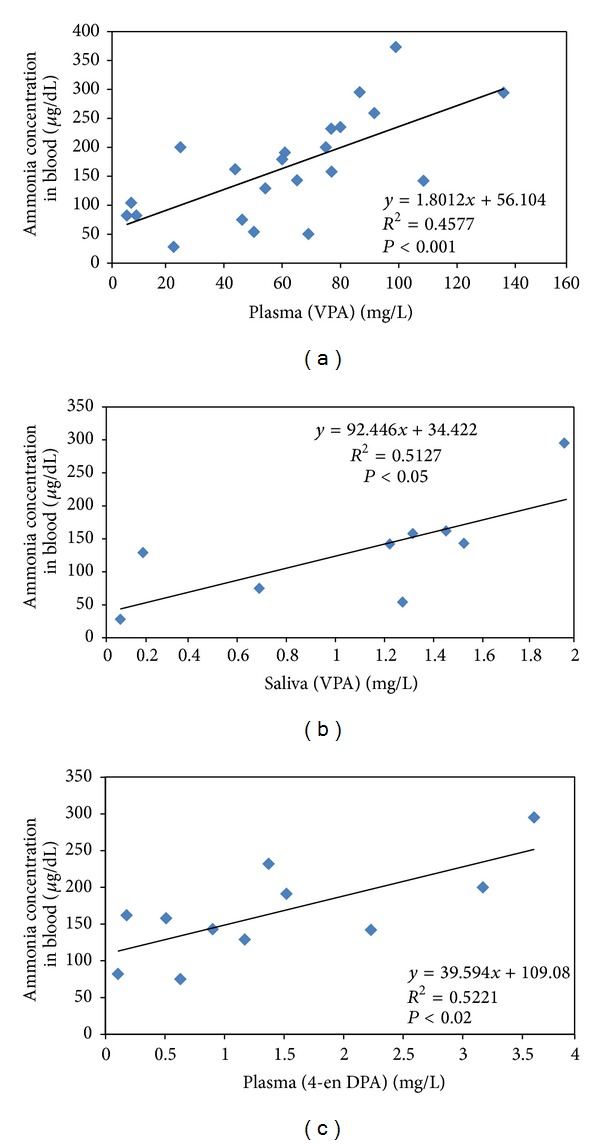
Plasma and saliva VPA concentrations and plasma 4-en VPA concentrations versus ammonia levels (a, b, and c, resp.).

Figures 2(a) and 2(b) show correlations between plasma and saliva levels of VPA versus ammonia concentrations in blood, respectively. As it can be observed, ammonia concentration increases with the increase of VPA concentration. Once more it becomes evident the higher correlation with the effect (toxic in this case) obtained from salivary samples (*R* ^2^ = 0.5127) in comparison to plasma (*R* ^2^ = 0.4577).  Figure 2(c) shows the correlation between 4-en VPA in plasma versus ammonia levels in blood showing that the formation of this metabolite, higher in accordance with higher VPA concentration, could really be one of the causes for the hyperammonemia. More formation of 4-en VPA due to *β*-oxidation impairment can result in inhibition of CPS, the first enzyme involved in ammonia detoxification through the urea cycle.

In the sixteen adult patients included in the study, some of them had more than one saliva and plasma VPA samples, whereas in some patients, saliva levels or 4-en VPA plasma concentrations could not be detected.

Although hyperammonemia was observed, most of the patients were asymptomatic and no hepatotoxicity was found in the individuals.

Since its introduction into clinical use, VPA has become one of the most widely prescribed drugs for epilepsy and lately for bipolar disorders. Despite the undisputed pharmacological importance due to its many action mechanisms already mentioned in the introduction section, hyperammonemia is still an unresolved problem with significant clinical and economic impact as it could turn into a factor to develop toxicity. Drug discontinuation is not the solution. So, our next aim was to study possible therapeutic actions. Valproic acid is commercially available in our country in many formulations: immediate-release (IR), delayed-release (DR), enteric-coated (twice-daily dose), and extended-release (ER, once-daily dosing) oral preparations. The conventional release preparations (IR and DR) are readily absorbed and reach a peak plasma concentration within 1 to 4 hours (important peak-trough fluctuations). In contrast, ER formulations are largely absorbed in the jejunum and take 3 to 5 hours or more to reach peak plasma levels. The main problem for ER (once-daily dosing) usage is the need to be administered in a higher daily dose due to a loss of bioavailability [32]. This is because valproic acid is carried to less favorable zones to be absorbed (more alkaline pHs) and at these pHs the ionized form predominates. In order to overcome the need of increasing ER daily dose, maintenance of the twice-daily regimen is suggested. Due to a significant decrease in the peak-trough fluctuation of plasma valproic acid levels, in comparison with the twice-daily dosing of conventional delayed-release formulations, concentration-dependent side effects would be prevented, also avoiding inefficacy of the treatment.

**Figure 3:**
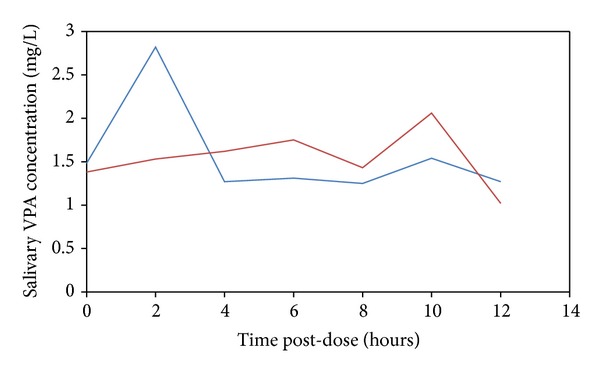
Salivary VPA curves after delayed-release VPA formulation (blue curve) and after extended-release VPA formulation (red curve).

Figure 3 shows the two salivary curves obtained after VPA morning intake (1000 mg) of a DR and ER formulation in the same patient. The blue curve is for the DR formulation and the red one is for the ER formulation.

Table 2 shows the pharmacokinetic parameters for the DR and the ER formulations. As it can be observed in Table 2, almost the same value was obtained for AUC_ss  0–12_ (19.1 mg · h/L and 18.1 mg · h/L for DR and ER, resp.) but a very different one for PTF (98.7 and 68.9, resp.) indicating that there are no loss in bioavailability and less pronounced peak-trough oscillations with the ER formulations. This is always expected with an ER formulation, but what is really interesting was that the ammonia level in blood after VPA DR formulation was 143 *μ*g/dL and 54 *μ*g/dL with the ER formulation (normal ammonia range in blood: 25–94 *μ*g/dL). The same efficacy was demonstrated in this patient with the two formulations but the toxic effect (hyperammonemia) was only observed with the conventional DR formulation. Although further studies are necessary to conclude that the extended release formulation is safer, it is a promising finding in order to give a therapeutic alternative to this problem.

Carnitine supplementation during VPA therapy in high-risk patients is now recommended by some scientific committees and textbooks, especially paediatricians [33–35] but there is no sufficient data available in the literature to support carnitine supplementation during VPA chronic administration. Carnitine thus appears essential to ensure proper metabolism of VPA. Carnitine prevents intramitochondrial accumulation of acyl-CoA by transforming acyl-CoA into acylcarnitine protecting the cell from toxic acyl groups. VPA in the cytosol is activated and links with reduced acetyl coenzyme A to form valproyl-CoA. Valproyl-CoA then crosses the outer mitochondrial membrane and by effect of palmitoyl carnitine transferase, valproylcarnitine is formed. This step is needed because the inner mitochondrial membrane is not permeable to acyl-CoA. In the mitochondrial matrix, another transferase transforms valproylcarnitine into valproyl-CoA, which is able to start the *β*-oxidation process.

Valproylcarnitine is excreted in urine. This is one of the mechanisms by which VPA produces carnitine depletion. However, because this excretion accounts for less than 1% in urine, it is unlikely that excretion of valproylcarnitine alone is sufficient to produce carnitine deficiency [36]. So perhaps, more than one mechanism is involved. Among others, a reduction in tubular reabsorption of both free carnitine and acylcarnitine and a reduction of endogenous synthesis of carnitine have been reported during VPA treatment. Moreover, valproylcarnitine inhibits the membrane carnitine influx transporter (OCTN2) expressed in kidney, thereby decreasing its renal tubular reabsorption [37].

Table 3 shows saliva and plasma VPA predose levels, 4-en VPA plasma level and ammonia level in the same patient before and after carnitine was added to the therapy. As it can be observed, ammonia level in blood was high before adding carnitine. No hepatotoxicity was found in this patient. After adding carnitine, not only a decrease in ammonia levels was found but also a decrease in VPA and 4-en VPA concentrations. This perhaps is indicating that L-carnitine supplementation descends ammonia levels because it reestablishes VPA altered kinetics. Once again, further studies have to be carried out in order to conclude on simultaneous administration of L-carnitine with VPA.

Interestingly, as it can be observed in Table 3, 4-en VPA/VPA ratio is higher before carnitine administration. This could be explained by a higher impact that the decrease of carnitine had on the metabolite (also a fatty acid) kinetics. The clearance of the metabolite is reduced as it cannot follow beta oxidation pathway and at the same time its bioavailability increases since VPA beta oxidation is reduced. More data comparing the metabolic ratio between patients with and without hyperammonemia is needed in order to confirm this observation. The ratio should be higher in patients with higher ammonia levels. In this way, the metabolic ratio could have an important diagnosis value.

## 4. Conclusions

Saliva can be an appropriate monitoring fluid to detect VPA inefficacy or toxicity as it is highly correlated with free drug. So it becomes not only a noninvasive, cheap, and easy to obtain fluid, but also a useful tool to detect therapeutic failure or undesirable effects. Moreover, citric stimulation is advisable for saliva collection.

Ammonia levels in blood depend on VPA and 4-en VPA concentration, so during VPA treatment ammonia in blood should be measured.

Further studies are necessary in order to conclude on therapeutic measures to be taken during VPA treatment. However, usage of VPA extended-release formulations administered every 12 hours or L-carnitine intake seem to be promising therapeutic measures to avoid this concentration-dependent adverse effect as hyperammonemia is.

## Figures and Tables

**Table 1:** VPA levels in plasma, plasma ultrafiltrate, and saliva in children under VPA monotherapy.

Dose (mg/day)	Plasma [VPA] (mg/L)	ult [VPA] (mg/L)	Saliva [VPA] (mg/L)	S/P	ULT/P	PB (%)	Salivary pHs
480	41.5	4.9	1.5	0.036	0.118	88.2	6.88
600	87.5	11.0	3.7	0.042	0.126	87.4	6.92
600	72.7	20.3	6.3	0.087	0.279	72.1	6.89
600	39.7	5.6	1.4	0.035	0.141	85.9	6.79
600	60.8	6.5	3.4	0.056	0.107	89.3	7.11
750	101.9	20.6	7.8	0.077	0.202	79.8	6.98
750	58.4	9.3	3.8	0.065	0.159	84.1	7.01
750	80.9	10.6	4.1	0.051	0.131	86.9	6.99
800	59.4	9.7	2.1	0.035	0.163	83.7	6.73
1000	65.6	9.1	2.8	0.043	0.139	86.1	6.89
1250	116.1	12.4	4.5	0.039	0.107	89.3	6.96
Means				**0.051**	**0.152**	**84.8**	**6.92**
S.D.				**0.018**	**0.050**	**5.0**	**0.10**

[VPA]: valproic acid concentration; ult: plasma ultrafiltrate; S/P: saliva/plasma ratio, ULT/P: plasma ultrafiltrate/plasma ratio; PB: protein binding; SD: standard deviation.

**Table 2:** Pharmacokinetic parameters in saliva after delayed-release and extended-release VPA intake.

Pharmacokinetic parameters	VPA DR	VPA ER
*C*max_ss_ (mg/L)	2.82	2.06
*C*min_ss_ (mg/L)	1.25	1.02
*T*max_ss_ (hours)	2	10
AUC_ss 0–12_ (mg·h/L)	19.1	18.1
*C*mean_ss_ (mg/L)	1.59	1.51
PTF	98.7	68.9

DR: delayed-release; ER: extended-release; PTF: peak-trough fluctuations; AUC_ss 0–12_: area under the curve from 0 to 12 hours; *C*max_ss_: maximum concentration at steady state; *C*min_ss_: minimum concentration at steady state; *T*max_ss_: time to obtain *C*max_ss_.

**Table 3:** VPA, 4-en VPA, and ammonia levels in a patient before and after carnitine supplementation.

	Patient (without carnitine)	Patient (with carnitine)
Plasma [VPA] (mg/L)	86.6	46.3
Saliva [VPA] VPA (mg/L)	1.9	0.68
Plasma [4-en VPA] (mg/L)	3.6	0.63
Ammonia concentration (*μ*g/dL)	295	75
[4-en VPA]/[VPA]	0.042	0.014

[4-en VPA]/[VPA]: metabolic ratio.
